# Near-Infrared Fluorescent Materials for Sensing of Biological Targets

**DOI:** 10.3390/s8053082

**Published:** 2008-05-08

**Authors:** Carrie L. Amiot, Shuping Xu, Song Liang, Lingyun Pan, Julia Xiaojun Zhao

**Affiliations:** 1 Department of Chemistry, University of North Dakota, Grand Forks, ND 58202, USA; 2 Coherent Light and Atomic and Molecular Spectroscopy Laboratory, College of Physics, Jilin University Changchun, Jilin, 130031 China

**Keywords:** Near-Infrared, Fluorescence, Metal-Enhancement, Nanomaterials, Bioimaging

## Abstract

Near-infrared fluorescent (NIRF) materials are promising labeling reagents for sensitive determination and imaging of biological targets. In the near-infrared region biological samples have low background fluorescence signals, providing high signal to noise ratio. Meanwhile, near-infrared radiation can penetrate into sample matrices deeply due to low light scattering. Thus, *in vivo* and *in vitro* imaging of biological samples can be achieved by employing the NIRF probes. To take full advantage of NIRF materials in the biological and biomedical field, one of the key issues is to develop intense and biocompatible NIRF probes. In this review, a number of NIRF materials are discussed including traditional NIRF dye molecules, newly developed NIRF quantum dots and single-walled carbon nanotubes, as well as rare earth metal compounds. The use of some NIRF materials in various nanostructures is illustrated. The enhancement of NIRF using metal nanostructures is covered as well. The fluorescence mechanism and bioapplications of each type of the NIRF materials are discussed in details.

## 1. Introduction

**Figure 1.**
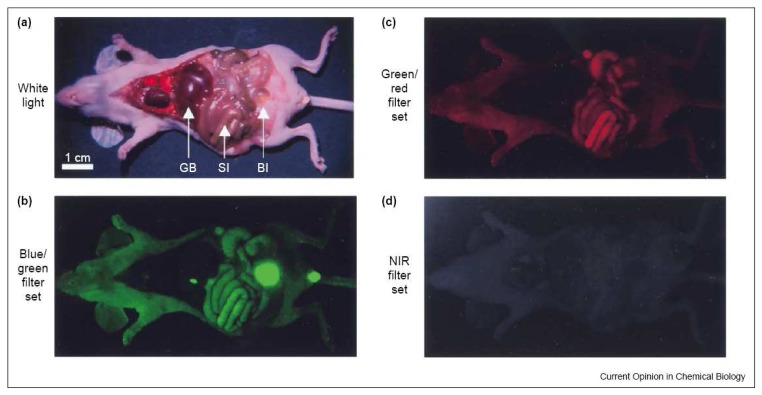
Autofluorescence observed in a mouse with various wavelength filter sets selected (GB: gall bladder, SI: small intestines, Bl: bladder) Reprinted with permission from [1] Copyright (2002) Elsevier.

Near-infrared fluorescence (NIRF) is an emerging branch within the field of fluorescence spectroscopy. Fluorescence spectroscopy has played an important role in analytical chemistry for over 50 years and continues to expand. The major advantage of fluorescence spectroscopy lies in a high signal to noise ratio and thereby achieving low detection limits. Recently, research interests on the near-infrared (NIR) portion of the electromagnetic spectrum (700 – 1000 nm) have increased tremendously. The studies focus on the development of novel NIRF materials and detection of various biological samples using these materials as fluorescent labeling reagents. The distinct features of NIRF over UV and visible region fluorescence include a lower background signal from biological samples and a deeper penetration of the radiation into biomatrices. The interfering background signal in the UV and visible region comes from autofluorescence of biological targets, which happens when tissues, proteins or other biomarkers fluoresce naturally. Thus, a high background signal appears in the detection of biological samples when visible fluorescence spectra are used. However, the absorption of the radiation and autofluorescence of these biosamples are at their lowest in the NIR region as demonstrated in Figure 1. Meanwhile, biological samples have less light scattering of NIR radiation, giving a deeper penetration of NIR emission to obtain inner structural information of biosamples. Because of these specific characteristics, one of the most prominent areas for NIR labels and sensors is *in vivo* imaging [1]. The penetration of the radiation can reach 2 – 5 cm into a sample. Therefore, the NIR region often is referred to as the “Biological Window”. Cheng *et al.* pointed out that “It is expected that NIR optical imaging will make a significant impact in disease detection and staging, drug development, and treatment assessment.”[2].

Theoretically, one major obstacle for the applications of NIRF is low signal intensity. Based on the quantum efficiency limitations of some radiation sources and detectors, longer wavelengths give lower intensity signals. Traditional NIRF dye molecules usually possess a low quantum yield, which also causes the diminished signal intensity. For example, indocyanine green (ICG), the most common NIRF dye which is used for the imaging of choroidal perfusion during angiography, only provide a quntumn yield of 1.2% [3]. In recent years, the development of a number of new intense and photostable NIRF nanomaterials along with the improvement of signal enhancement techniques for NIRF materials make NIRF measurements feasible.

Four categories of NIRF materials are currently used: 1) fluorescent dyes, 2) quantum dots, 3) single-walled carbon nanotubes, and 4) rare earth metal reagents. Of the four types of NIRF materials, organic fluorescent dyes are traditional and frequently used. Among different types of organic dye molecules, the cyanine family of dyes is the most commonly used in sensing of biological targets due to their biocompatibility. Quantum dots (QDs) are the second most common NIRF material in biological studies. Most QDs are nanoscale semiconductors with tunable emission wavelengths. By increasing the size of a QD, the emission wavelength is increased as well. In addition to the two popular NIRF materials above, single-walled carbon nanotubes (SWNTs) are growing as a new category of NIRF probes. When individually suspended in solution they have proven to be successful bioprobes [4-5]. The fourth type of NIR material is rare earth metal compounds. But they are seldom used in the biological applications. Instead they usually are doped in glass for fluorescence lifetime studies, or for making new types of lasers.

This review covers recent developments of the four types of NIRF materials. The fluorescence mechanism of each type material is discussed along with their optical properties. The applications of these NIRF materials in biological field are covered as well. The detected biological targets include metal ions [6-9], small molecules [10], DNAs [4-5,11], proteins [2,12-14], amino acids [15], bacteria [16] and tumors [17-19].

## 2. NIRF Dye Molecules

Near-infrared fluorescent dyes are the traditional NIRF materials. Their molecular structures are highly conjugated and have a lower energy gap between the ground and excited states than visible region fluorescent dyes. So far three main types of NIRF dyes are commonly used including cyanine (Cy) dyes [17,20], squaraine dyes [21-24], and thiazine and oxazine dyes [25]. Recently a novel class of conformational restricted aza-dipyrromethene boron difluoride (aza-BODIPY) dyes [26] has been synthesized. This dye shows a high chemical stability and photostability and may become a promising NIRF reagent in the near future. In addition, porphyrin array dyes [27-29] along with metal phthalocyanine dyes [30] provide both visible and NIR region absorbance and fluorescence.

The fluorescence mechanism of most NIR dyes is based on electron transitions between molecular electronic states. An electron is promoted from the ground state to an excited state when the molecule absorbs a photon from a radiation source. Relaxation occurs to the lowest vibrational energy of each excited state. If the electron is not at the first excited state, internal conversion can occur, followed by further relaxation to the lowest vibrational energy of the lowest excited state. From this point, energy is released as the electron returns to the ground state. This can occur by nonradiative emission as heat or radiative emission as fluorescence. The energy change of excitation (Δ*E*_ex_) is generally larger than the energy change of emission (Δ*E*_em_), producing a longer wavelength for the emission radiation than the excitation.

The physical and chemical properties of NIR dyes are adjustable for different applications through chemical modification of the dye molecules. These properties include solubility of the dye in aqueous solutions, the excitation and emission wavelengths, the biocompatibility in a given matrix, the binding ability of the dye to the probe for a single analyte, *etc.* The modifications provide the dye molecules much broader applications in the biological field. Several important modification methods are discussed below.

### 2.1 Improving Water Solubility and Reducing Aggregation

**Figure 2.**
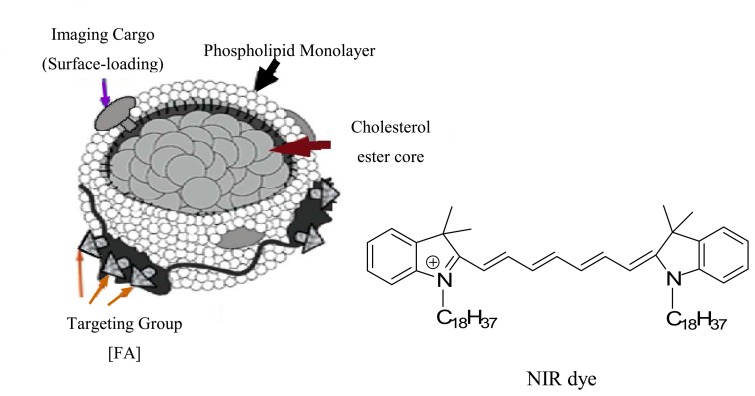
Schematic diagram of a hydrophobic NIR dye molecule assembled into a natural cholesterol nanostructure. Reprinted with permission from [31]. Copyright (2007) American Chemical Society.

To effectively use NIRF dyes for sensing of biological samples, a hydrophilic nature is usually essential. However, a number of NIRF dyes are not water soluble due to their highly conjugated structures. Thus, suitable modification of these NIR dyes is needed prior to their bioapplications. Gaining solubility in aqueous solutions is often achieved by linking sulfonate groups to the dye structure [2,8]. The presence of highly hydrophilic sulfonate groups makes the dye molecules water soluble. Alternatively, the dye molecules can be assembled inside a hydrophilic shell that has a hydrophobic inner layer. For instance, Chen *et al.* demonstrated that a dye does not have to be water soluble to be biocompatible [31]. They imbedded the hydrophobic NIRF dye molecules into the phospholipids monolayer of low density lipoprotein (Figure 2). The cholesterol ester core absorbed the dye and functioned as a carrier to deliver the dye into bio-targets.

Modifications may change the molecule emission wavelength and reaction ability. The potential changes in the molecule activity must be considered. As larger ligands are employed, more steric interactions occur between the dye and the probe. The modification can reduce chemical reaction efficiency when the dyes are used for sensitive determinations. The sulfonate groups bring negative charges to the dye molecules. The large number of negative charges will hinder some binding between the dye and the negatively charged bioanalytes [1].

In addition to the hydrophobicity problem, aggregation is another major drawback of NIRF dye molecules. The dye molecules can easily aggregate in aqueous solutions, resulting in low fluorescence intensities and blue-shift of the absorption peak. Two effective methods can prevent the aggregation. One is to mix a certain amount of water soluble organic solvents into the aqueous solution to dissolve the dyes. Flanagan *et al.* have shown improved water solubility by dissolving cyanine type dyes in a buffer solution containing 40% methanol. The absorption peak at 650 nm (aggregates of dyes) reduced and the absorption peak from monodispersed dye molecules at 760 nm became narrower and intense [16]. The second way to prevent aggregation is to conjugate the dye molecule with a long “tail” of nucleotides, such as a propynyl amino-modified nucleotides (7-(3-Amino-1-propynyl)-2′,3′-dideoxy-7-deazaadenosine 5′-triphosphate (ddATP) and 7-(3-Amino-1-propynyl)-2′,3′-dideoxy-7-deazaguanosine 5′-triphosphate (ddGTP) [16]). The UV-Vis absorbance spectrum of the conjugates proves no aggregation in aqueous solution after these tails are linked to the dyes. In addition to the nucleotides, other large biomolecules, such as human serum albumin, have a similar function when they are linked to the NIR dye molecules [32-33].

### 2.2 Separation of Excitation and Emission Bands

A challenge with many NIRF dyes is that the excitation band and the emission band significantly overlap. Only when the two peaks are discrete, can they be most effectively used for the detection of targets. A longer wavelength gap between the two maximum values indicates that a greater sensitivity can be attained. So far, two approaches can separate the emission and excitation peaks effectively.

The first approach is to change the molecular structure of the dye slightly, yet not to change the binding properties. A minor modification induces a blue shift in the excitation wavelength. This shift has been obtained by Peng *et al.* [7] and Kiyose *et al.* [8]. In both cases the center of the dye, where the spacer and probe were attached, was altered to have a nitrogen atom rather than oxygen, sulfur or any other atoms. Due to an excited-state intramolecular charge transfer, a slight blue shift of emission band occurred, while a significant blue shift for the excitation band. As a result, the difference between excitation and emission peaks increased from 30 nm to 150 nm [7].

**Figure 3.**
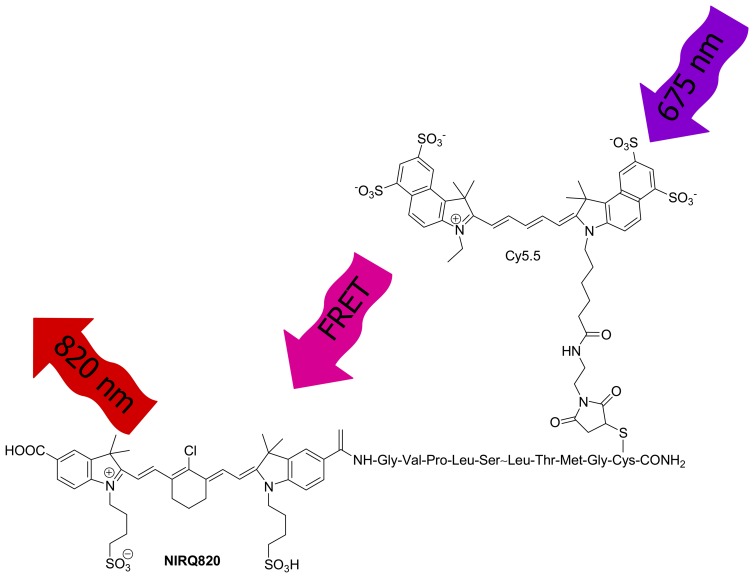
FRET occurs between Cy5.5 and NIRQ820 [14]. Reprinted with permission from [14]. Copyright (2004) American Chemical Society.

Modification of dye molecular structures is not always feasible. Alternatively, two dyes can be used as one effective NIR dye if one dye emission overlaps with the other excitation. Based on fluorescence resonance energy transfer (FRET), one dye can be a donor and the other is an acceptor. The excited state of the donor transfers energy to the acceptor by a nonradiative, long-range dipole-dipole coupling mechanism. This transfer requires the distance between the donor and acceptor to be close enough, typically less than 10 nm [34]. For example, the emission band of Cy5.5 overlaps with the excitation band of NIRQ820 [14]. When the two dye molecules closely exist, the excitation wavelength is 675 nm provided by Cy5.5 and the emission wavelength is 829 nm given by NIRQ820 (Figure 3). The emission and excitation peaks are completely separated.

### 2.3 Fluorescence Lifetime of NIRF Dye Molecules

Fluorescence lifetime is an important property of dye molecules. A longer lifetime gives excited electrons a greater possibility to release energy through non-radiation. The lifetime of NIRF dyes can be changed upon modifications of dye molecular structures. It has been reported that the NIR dye molecules containing different heavy atoms (halogen) can alter their fluorescence lifetime. The lifetimes of these dyes vary with the identity of the halogen substitution near the center of the dye. The heavier halogen atom gives the longer lifetime. An average variation within the same dye series is about 35 ps [12]. The experimental results from flash photolysis techniques indicate that the modification of dyes using a heavy-atom increases the efficiency of electron crossing into the triplet state. The heavier atom shows a larger rate of intersystem crossing. Thus, the effect of modification of molecular structures on NIRF dye lifetime should be considered prior to any modifications. Increased lifetime results in lower fluorescence quantum yield, and thus lower signal intensity.

### 2.3 Fluorescence Lifetime of NIRF Dye Molecules

Fluorescence lifetime is an important property of dye molecules. A longer lifetime gives excited electrons a greater possibility to release energy through non-radiation. The lifetime of NIRF dyes can be changed upon modifications of dye molecular structures. It has been reported that the NIR dye molecules containing different heavy atoms (halogen) can alter their fluorescence lifetime. The lifetimes of these dyes vary with the identity of the halogen substitution near the center of the dye. The heavier halogen atom gives the longer lifetime. An average variation within the same dye series is about 35 ps [12]. The experimental results from flash photolysis techniques indicate that the modification of dyes using a heavy-atom increases the efficiency of electron crossing into the triplet state. The heavier atom shows a larger rate of intersystem crossing. Thus, the effect of modification of molecular structures on NIRF dye lifetime should be considered prior to any modifications. Increased lifetime results in lower fluorescence quantum yield, and thus lower signal intensity.

### 2.4 Bioapplications of NIRF Dye Molecules

NIRF dye molecules have demonstrated great potential as a highly sensitive fluorescent labeling reagent for *in vivo* and *in vitro* bioimaging. The biological targets include tissue, cells, proteins, DNAs, metal ions, *etc.* Usually, the dye molecule is linked to a probe that can specifically bind to the target. The probe can be an antibody, a single strand DNA or a ligand. To link the dye molecules to the probes, the dye molecules are modified with various functional groups, such as amine group [8,16], succinimidyl ester group (which is converted into a carboxyl group in the reactions) [16] or an isothiocyanate group (ITC) [12,16]. The functional groups allow the dye molecules to attach to biomolecules and other small molecules. Hammer *et al.* [16] have compared the susceptibility of three Cy dyes with the succinimidyl ester and isothiocyanate groups when they conjugated with amino-functionalized dideoxynucleotide triphosphates. The succinimidyl ester groups are very sensitive to hydrolysis, but the isothiocyanates are not. The alkylisothiocyanate dye (those with an alkyl chain spacer) shows better conjugation efficiency to highly negatively charged nucleotides than an ITC group, with a very short spacer next to the dye molecule.

## 3. NIR Luminescent Quantum Dots

Quantum dots (QDs), also called nanocrystals, are a special class of materials known as semiconductors. Currently, they are composed of periodic groups of II-VI, III-V, or IV-VI elements. The size of QDs is in the range of 2-10 nanometers (10 - 50 atoms) in diameter. As the size of the QD changes, there is an obvious difference in the emission wavelength [35]. Thus, simultaneous detection of multiple targets at different emission wavelengths with a single excitation wavelength is a remarkable feature of QDs [36]. As opposed to many organic dyes, QDs are highly photostable. Normally, the QDs are formed from a number of heavy metal inorganic compounds, such as PbSe, InAs, CdSe, and CdTe *etc.* However, a majority of these compounds are toxic to living systems. To reduce the toxicity and increase biocompatibility, functionalization of the surface of QDs is necessary by postcoating a biocompatible shell, which is commonly done by various organic compounds.

### 3.1 Luminescence Mechanism of QDs

The energy level range of QDs is significantly different from that of bulk semiconductors. In bulk semiconductor materials, the energy levels are a continuous series. When the sizes of semiconductor crystals are reduced to the Bohr exciton radius, which is the mean distance between the electron and the hole, its energy levels become discrete because of the quantum confinement effect. Semiconductor crystals with sizes in this scale are referred to as quantum dots. Quantum confinement effect widens the HOMO and LUMO gap of QDS and causes the electronic structure and photophysics to be greatly changed compared with that of bulk materials. The width of this energy band gap determines the absorption band edge of QDs. Thus the absorption and luminescence properties of QDs strongly depend on the size of QDs [37]. As the size increases or decreases, both the absorption and luminescence peaks undergo red or blue shifts in respect to the diameter.

The indirect bandgap semiconductors are special since their HOMO and LUMO do not match. If the electron is recombined with a photon while being excited, it will be promoted to the LUMO through an indirect path. This lower bandgap provides emission in the NIR region. Silicon QDs are an example of indirect bandgap semiconductors. However, not all the excited modes of silicon QDs are the indirect bandgap transitions. The surface passivants of semiconductor nanocrystals can influence their photoluminescence. Oxygen can directly interact with Si orbitals, which substantially changes the HOMO and LUMO as compared with hydrogen passivation [38]. The 1 to 2 nm H-passivated nanocrystals tend to emit blue/violet light for dipole-allowed transitions whereas core/shell oxide-passivated nanocrystals emit yellow-red light for dipole-forbidden transitions.

A few recent works reported that noble metal nanoclusters with a ligand monolayer can behave as QDs. One example is an Au nanocluster that was composed of several tens or hundreds of atoms with ligands protecting the surface. The size of the Au nanocluster is less than 3 nm. Unlike the above semiconductor nanocrystals, the luminescence mechanism of these monolayer-protected Au clusters (MPCs) is not based on the HOMO-LUMO energy gaps. Rather, the luminescence mechanism of the Au-nanocluster is based on the metal-to-molecule transition from the Au cluster to the ligands [39].

### 3.2 Structures of NIRF QDs

A number of core-shell NIRF QDs has been reported, such as CdHgTe/ZnS nanocrystals [34,40-42], CdHgTe nanorods [43], CdZnTe [44], InAs/CdSe/ZnSe core/shell1/shell2 (CSS) structure [45], silicon QDs [46-48], *etc.* The core-shell structure of QDs is popular because it provides a higher fluorescence quantum yield. The core is composed of the wider band gap composition, while the shell is composed of the narrower band gap composition. This core-shell structure can improve the efficiency of radiated transition and restrict the irradiated transition. For example, an InAs/CdSe/ZnSe core-shell-shell (CSS) structure yields remarkably high fluorescence quantum yield and gives a high photostability covering the entire NIR range from 800 nm to over 1,600 nm [45].

The fluorescence of QDs is strongly affected by the surface ligands. As mentioned above, the core-shell structure restricts the irradiated transition; the same is true with the ligand on the QD surface. Murray *et al.* found that the near-infrared photoluminescence of Au_38_ and Au_140_ MPCs is intensified with exchange of nonpolar ligands by more polar thiolate ligands. The effect includes more ligands: thiophenolates with a variety of *p*-substituents; alkanethiolates *ö*-terminated by alcohol, acid, or quaternary ammonium groups; and thio-amino acids [39].

### 3.3 Bioapplications of NIRF QDs

Previously, QDs were limited in their applications for bio-labeling targets because of their hydrophobic nature. With the development of various new syntheses and surface modifications, QDs can be prepared in aqueous solution or suspended in water by the phase transfer method. This surface treatment also minimizes the quenching effect that is generally observed with QDs in aqueous solution. Wiess *et al.* [34] have developed a hybrid approach to synthesize CdHgTe/ZnS core/shell NIRF QDs in water phase. The quantum yields of the QDs are 20-50% in the NIR region.

**Figure 4.**
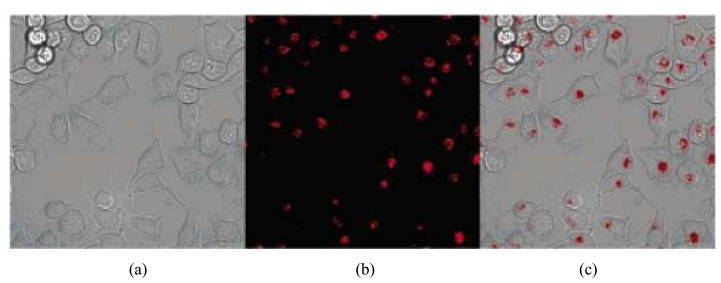
Cell staining of NIRF CdHgTe/ZnS QDs in micelles on live P815 mast cells. (a)Bright field image of cells, (b) confocal fluorescence microscopy image, and (c) merged image. Detection wavelength range is 650-750 nm. Reprinted with permission from [34]. Copyright (2004) American Chemical Society.

The hydrophilic property provides QDs a wide variety of applications in biological and biomedical fields. The single QDs can be traced in living cells for *in vivo* and *in vitro* imaging of biological process [49-50]. So far, a large number of research papers have been published in this area. Figure 4 shows a typical example of imaging cells using QDs as a fluorescent labeling reagent. The living P815 mast cells were incubated with phospholipid micelle coated QDs overnight and thus the cells were labeled by the QDs [34].

**Figure 5.**
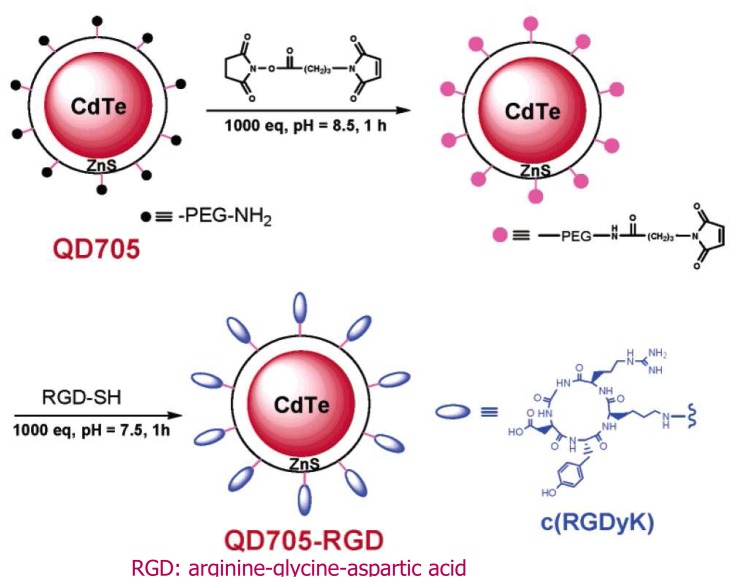
Core-shell structure of QDs with functionalized surface to bind to a specific target. Reprinted with permission from [19]. Copyright (2006) American Chemical Society.

Figure 5 shows another example of the application of the typical CdTe/ZnS core/shell QDs for *in vivo* targeting and imaging of tumor vasculature [19]. The amine functionalized poly(ethylene glycol) (PEG) was postcoated on the QD surface to link (RGDyK) probe to the QDs for bioimaging.

## 4. Single-walled Carbon Nanotubes

Carbon nanotubes were first reported in 1976 [51]. However, it was not until 1991, when Iijima clarified the atomic structure and character of single-walled carbon nanotubes (SWNT) and multi-walled carbon nanotubes (MWNTs), that the real growth began in this field [52]. The majority of the research has focused on mechanical, thermal, and electronic properties of carbon nanotubes. In 2002, O'Connell *et al.* [53] first reported the band gap fluorescence from an individual SWNT. Afterwards, the interest in the optical properties of carbon nanotubes has increased. The bioapplications of the carbon nanotubes have demonstrated that SWNTs are promising contrast agents and fluorescent labels for bioimaging [54-55].

### 4.1 Structure of SWNTs

**Figure 6.**
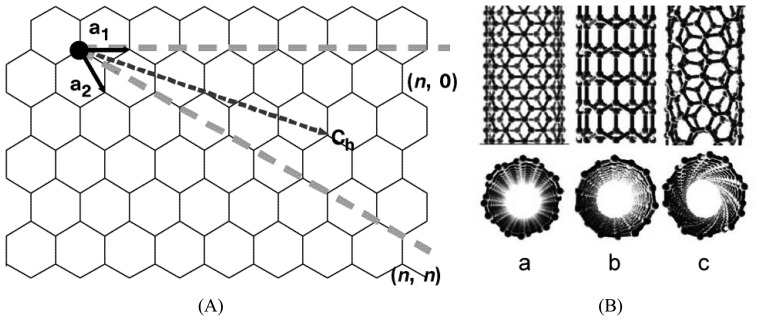
(A) The chiral vector of a carbon nanotube in a two-dimensional graphene. Reprinted with permission from [59]. Copyright (1998) Nature Publishing Group; (B) Molecular models of single-walled carbon nanotubes: armchair (a), zigzag (b), and chiral (c). Reprinted with permission from [52]. Copyright (2002) AAAS.

Carbon nanotubes are entirely composed of carbons in *sp^2^* state [56]. SWNTs are single tubes that are approximately 1 nm in diameter and 1∼100 μm in length. Its length to diameter ratio is in the range of 100∼1000 [57]. Therefore, carbon nanotubes are an ideal one-dimensional material [58]. The structure of a SWNT can be conceptualized as a one-atom-thick layer of graphite (called graphene) wrapped into a seamless cylinder. The wrapping style of the graphene sheet is represented by a pair of indices (*n*,*m*) called the chiral vector. Figure 6A shows that the chiral vector is defined as C_h_ = *n*a_1_ + *m*a_2_. The integers *n* and *m* denote the number of unit vectors along two directions in the honeycomb crystal lattice of graphene. T denotes the tube axis and a_1_ and a_2_ are the unit vectors of graphene in real space. Three types of nanotubes are possible: armchair, zigzag and chiral, depending on how the two-dimensional graphene sheet is “rolled up.” If *m* = 0, the nanotubes are called “zigzag,” and when *n* = *m*, they are called “armchair.” At any other angle, nanotubes are called “chiral.” (Figure 6B) [57-58].

### 4.2 Fluorescence Mechanism of SWNTs

The unique structure of carbon nanotubes results in their special optical properties. The distinctive NIRF arises from the one-dimensional direct electronic band gap. The band gap size is inversely related to the SWNT diameter. A narrow excitation peak in the visible region (500-600 nm) produces an intense fluorescence in the NIR region [53]. Their fluorescence lifetime is usually very short (< 2 ns) compared to other NIRF materials. The short lifetime eliminates non-radiative deactivation and thus gives SWNTs high fluorescence quantum yields. The fluorescence of SWNTs can be quenched when they are aggregated in bundles. This is the reason that MWNTs have no fluorescence signals. A MWNT is composed of multiple SWNTs assembled within one another in a large tube. Thus, to achieve high fluorescence intensity using SWNT probes, the concentration of SWNTs should be carefully optimized, along with modifying the surface for increased solubility [60-61].

### 4.3 Bioapplications of SWNTs

**Figure 7.**
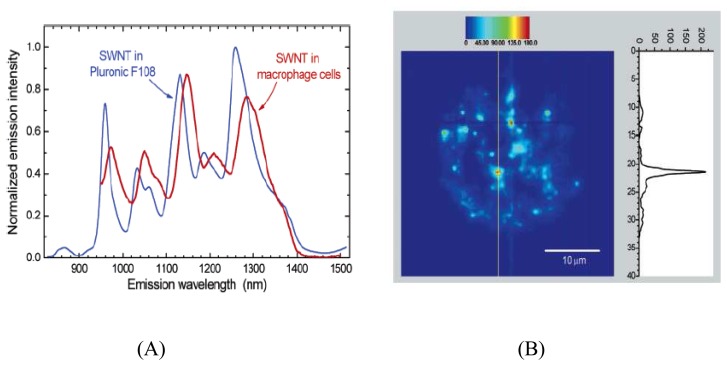
(A) SWNTs emission spectra in an aqueous suspension and in macrophage cells; (B) Fluorescence image of one macrophage-like cell incubated with SWNTs [62]. Reprinted with permission from [62]. Copyright (2007) American Chemical Society.

Due to the excellent photostability and high fluorescence quantum yield, the SWNTs have been applied as optical sensors for *in vivo* imaging. Cherukuri *et al.* used NIRF SWNTs to image phagocytic cells [62]. Low concentrations of nanotubes in biological specimens were selectively detected using a spectra-fluorometer and a fluorescence microscope modified for the NIR region. Cultured mouse peritoneal macrophage-like cells were incubated in growth mediums containing different concentrations of SWNTs. Analyses of the washed cells showed that the ingested nanotubes remain fluorescent (Figure 7A) and can be imaged through a NIRF microscope at wavelengths beyond 1100 nm (Figure 7B).

Recently DNA wrapped SWNTs have been used to make an *in vivo* glucose sensor through immobilization of glucose oxidase onto the SWNTs [3]. Another sensor employed a DNA wrapped carbon nanotube with a magnetic iron oxide nanoparticle at one end [4]. This design allows the sensor to trace both NIRF signal and magnetic resonance imaging (MRI). It is expected that more bioapplications of SWNTs will appear in the near future.

## 5. Lanthanide Rare Earth Metal Compounds

Several lanthanide (III) (Ln^3+^) compounds are intrinsic NIRF materials [63-65]. However, the low absorbance coefficients (ε ≤ 1M^-1^ cm^-1^) make the rare earth metal ions difficult to be pumped to the excited state. Thus, this type of NIRF materials is usually employed for fundamental studies [66-68].

### 5.1 Fluorescence Mechanism of Rare Earth Metal Compounds

The low absorbance coefficients of Ln^3+^ compounds indicate that the excitation of Ln^3+^ electrons is difficult. Moreover, once the electrons are excited, they are particularly prone to nonradiative, vibrational deactivation. To assist electrons in reaching the excited state, some organic ligands are coordinated to the metal ions. The selection of the organic ligands is strict. If the ligands contain a number of high energy oscillators, such as C-H and O-H, quenching of the fluorescence more likely occurs. In general, polyfluorinated ligands can reduce the quenching effect. Once a suitable organic ligand is coordinated, the complex shows efficient NIR emission under UV excitation. In addition, the organic ligands can stabilize the Ln^3+^ ions and decrease their aggregation.

The Ln^3+^ emission lines are from multiple energy levels. It is possible for Pr^3+^ ions to emit from three energy levels, ^3^P_0_, ^1^D_2_ and ^1^G_4_. In fact the peaks observed all come from ^1^D_2_ level, relaxing to different ground states, ^1^D_2_ → ^3^F_2_, ^1^D_2_ → ^3^F_4_ and ^1^D_2_ → ^1^G_4_. In contrast to Pr^3+^ and the emission of most other Ln^3+^ ions, Yb^3+^ ions only have emission possible from a single excited energy level, ^2^F_5/2_. The characteristic emissions allow for rare earth metal compounds to be used as bioprobes and protein conjugates for chiral assays of biological structures and medical diagnostics [69].

### 5.2 Applications of NIRF Rare Earth Metal Compounds

Lanthanide metal ions are frequently used in the studies of various mechanisms, including redox reactions and fluorescence lifetime. Meanwhile, they are employed to make laser materials and as dopants to fabricate specific glass or crystals. The fundamental studies are based on up-conversion of fluorescence when the elevated electrons are further excited to a higher energy level and then emit fluorescence as they return to the ground state. The two photon process gives a larger energy gap, and a lower emission wavelength, which provides an efficient vehicle for fundamental study.

The engineering of rare earth metal compounds produces a number of new NIRF materials that demonstrate promising applications in bioanalysis. For example, Yb^3+^ and Tm^3+^ were co-doped into a NaYF_4_ nanocrystal [70]. The produced new nanocrystals have an excitation band at 980 nm and emission bands at 474 nm and 800 nm. The nanocrystals can be used as labels for sensitive determinations or *in vivo* imaging of biological samples. Furthermore, NIRF properties of rare earth metal compounds can advance laser features when they are doped into laser materials. Recent reports showed that the laser intensity, thermal stability, and fluorescence lifetime have been greatly improved by doping Tm^3+^ and Yb^3+^ in oxyfluoride silicate glasses [66] and neptunyl(VI) [71]. It is expected that more applications of rare earth metal compounds in biological field will be feasible in the future.

## 6. Assembly of NIRF Materials into Nanoscale Matrix

The sensitivity of detection of biological targets using NIRF molecules is limited by the small number of NIRF molecules attached to a single target. Usually this number is from 1 to 5. To improve the sensitivity, a large number of NIRF molecules are assembled into a nanoscale matrix to form an intense fluorescence nanoprobe. Besides the strong fluorescence signal, the NIRF nanomaterials exhibit excellent photostability because the nanomatrix protects dyes from environmental oxygen. Various types of nanomatrices have been reported. In this review we cover only a few which possess following characteristics: 1) transparent and non-fluorescent in the visible and NIR region; 2) biocompatible; 3) inert to the NIRF materials and surrounding environment; 4) low toxicity. Based on current experimental results, silica, carbonate and some polymers fit these conditions.

### 6.1 Silica-based NIRF Nanomaterials

The inert silica nanoparticles have low toxicity to living cells [72]. Its negatively charged matrix provides numerous electrostatic binding locations for a wide variety of positively-charged dye molecules. Thus, silica provides NIRF an excellent matrix to form nanomaterials.

**Figure 8.**
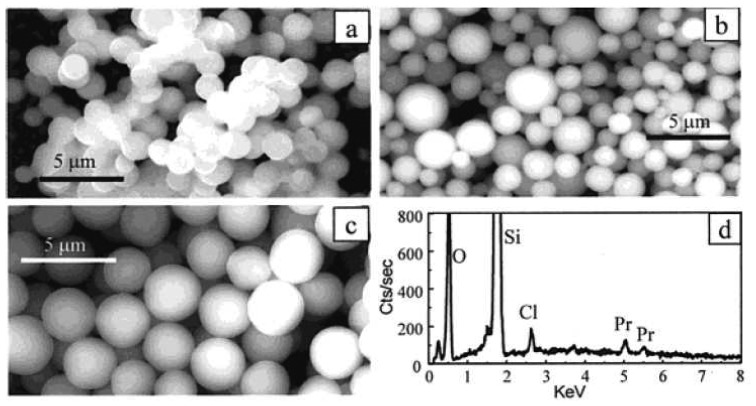
Scanning electron microscopy images. (a) Silica microparticles without dopants form necks between particles, (b) Pr-doped poly-dispersed silica particles, (c) low mono-dispersed Pr-doped microparticles, and (d) an energy dispersive X-ray (EDAX) spectrum of the mono-dispersed Pr-doped microparticles shown in (c). Reprinted with permission from [73]. Copyright (2001) American Chemical Society.

Moran *et al.* used acetic acid to catalyze the hydrolysis and condensation of tetraethyl orthosilicate (TEOS) in the presence of LnCl_3_·6H_2_O to produce lanthanide-doped silica microspheres as shown in Figure 8 [73]. The fluorescence spectra of Pr^3+^ and Er^3+^ remained the same after they were doped in the silica nanomatrix. A series of NIR dyes with lanthanide complexes were successfully doped into silica-poly(ethylene glycol) sol-gel hybrid material [74]. The complexes are very stable in the sol-gel matrix.

**Figure 9.**
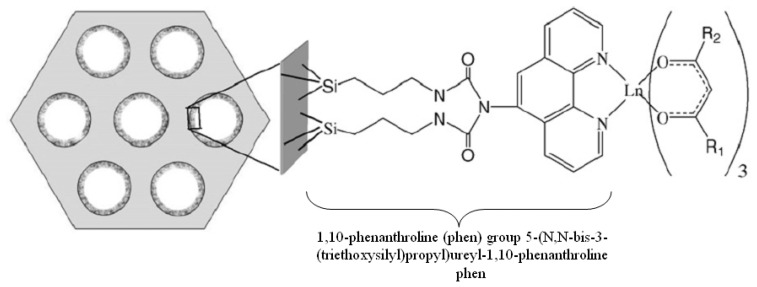
Expected structure of the Ln(hfth)_3_phen–M41 (Ln = Er, Nd, Yb, Sm) and Pr(tfnb)_3_phen–M41 mesoporous materials. hfth = 4,4,5,5,6,6,6-heptafluoro-1-(2-thienyl)hexane-1,3-dionate; tfnb = 4,4,4-trifluoro-1-(2-naphthyl)-1,3-butanedionate. Reprinted with permission from [69]. Copyright (2007) Elsevier.

In addition to silica nanospheres and sol-gel matrix, some materials with nano-sized pores can be used to incorporate the NIRF materials, such as mesoporous silicate-based materials. Sun *et al.* [69] covalently immobilized ternary lanthanide (Er^3+^, Nd^3+^, Yb^3+^, Sm^3+^, Pr^3+^) complexes to the phen functionalized mesoporous MCM-41 (Figure 9). The ligands around the lanthanide ions (including hfth/tfnb and phen ligands) could efficiently transfer the absorbed energy to the lanthanide ions. NIRF from lanthanide ions (Er^3+^, Nd^3+^, Yb^3+^, Sm^3+^, Pr^3+^) have been obtained in the region of 1300-1600 nm. The highly ordered hexagonal channel structures and uniform tunable pore sizes of MCM-41 mesoporous material open a new field to orderly composite materials for lasers and optical amplification.

### 6.2 Carbonate and Polymer-based NIRF Nanomaterials

Carbonate and polymers are also used as nanomatrices for encapsulation of NIRF materials. Gaponik *et al.* [42] prepared a NIRF microcapsule by adsorbing water-soluble Cd*_x_*Hg_1-_*_x_*Te nanocrystals in a hydrophilic carbonate core via polyelectrolytes. The produced capsules can be used as fluorescent labels for imaging of cells and tissue.

**Figure 10.**
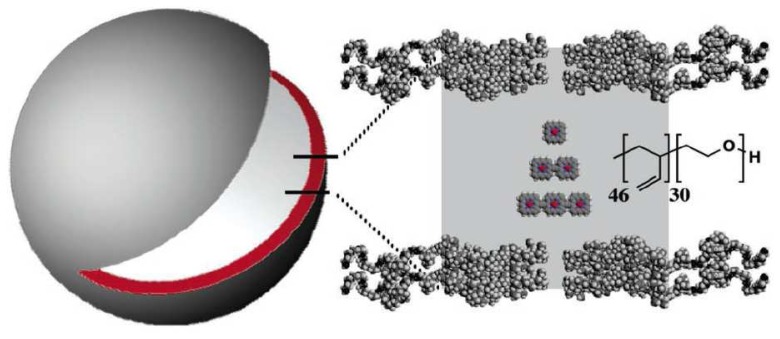
(A) Schematic diagram of polymersomes incorporating mono-, bis-, and tris(PZn)-based chromophores. Reprinted with permission from [29]. Copyright (2005) American Chemical Society.

Biocompatible polymers are attractive matrix for functionalization of biomaterials. Ghoroghchian *et al.* [29] reported a NIR emissive polymersomes with a broad fluorescence wavelength modulation. A variety of ethyne- and butadiyne-bridged (PZn)-in polymersomes were incorporate to supermolecular fluorophores (Figure 10). Controlling polymer-to-fluorophore noncovalent interactions finely tunes the photophysical properties of the encaplsuted supramolecular materials. The developed NIRF nanostructure was used to label dendritic cells (DCs) [75]. A lower detection limit of 5,000 DCs was obtained.

The polymer and silica can form a hybrid matrix for encapsulating dye molecules. Recently, Yu *et a1.* synthesized a stable, non-liposomal silica/polymer microcapsules [Poly(allylamine hydrochloride), PAH] [76]. The microcapsule can absorb a large number ICG. The final ICG content of 23% by weight was obtained with a minimal leakage compared to other ICG-doping particles. The ICG-containing capsules were active for NIR laser-induced heating, and were more photostable than free ICG.

Besides the inorganic and polymer nanoparticles, some biomolecules are suitable nanocarriers for delivering of NIRF materials to targets. One example is the lipoprotein nanoparticles (Figure 2) [31]. The low-density lipoproteins (LDL) particles are biocompatible, biodegradable, and non-immunogenic. Their size can be precisely controlled (∼22 nm) by its apoB-100 component through a network of amphipathic R-helix proteinlipid interactions. Chen *et al.* developed a ligand-modified NIRF dye functionalized LDL nanoparticle. The LDL nanoparticles enabled the first *in vivo* validation of the LDL rerouting principle. This was also the first *in vivo* application of the bio-nanocarrier for enhanced optical imaging of cancer cells in living animals.

## 7. Metal Enhancement of NIRF

The high signal intensities, low detection limits, and fast response time are critical for sensitive determinations of trace amounts of biological targets. Thus, the high fluorescence intensity is desirable for all label reagents. However, the signal intensity of NIRF materials is intrinsic and limited by its maximum value. To raise the limit of their intrinsic intensities, signal amplification is required. Noble metal nanostructures (e.g. Au, Ag) can enhance the fluorescence intensities of NIRF molecules which exist in their electromagnetic field. This phenomenon is called surface plasmon resonance enhancement. Based on the radiation mechanism of photoactive material, the surface enhancement can be further divided into surface/metal-enhanced fluorescence [77] and surface/metal-enhanced chemiluminescence, *etc.*

### 7.1 Enhancement Effect of Metals on Fluorophores

The fluorescence enhancement of metals on dye molecules is determined by metal nanostructures. In nanodimension, the collective oscillations of metallic free electrons are limited by the nanostructure boundaries, and thus form the surface plasmon waves along the boundaries. The surface plasmon wave resonates with the incident wave at an optimal condition, which results in the greatest absorption of the incident light. The oscillation wave affects the nearby fluorophores in several aspects.

First of all, on the surface of metal nanostructure, the occurrence of surface plasmon resonance leads to a strongly enhanced absorption of the incident light. When the surface plasmon resonance band of metal nanostructure overlaps with the excitation of the fluorophores, the energy is transferred from the metal to the fluorophore so that the possibility of excitation of the dye molecule is increased. Thus, more ground state electrons of dye molecules can be transferred to the excited state and produce a higher fluorescence signal. Secondly, the metal nanostructure can change the radiative deactivation rate (Γ) of the fluorophores. Furthermore, the fluorescence lifetime (τ) and the quantum yield (*Q*) are changed based on the Equation (1) and (2) [32]:

(1) $$Q=\frac{\Gamma }{\Gamma +k_{nr}}$$

(2) $$\tau =\frac{1}{\Gamma +k_{nr}}$$

Here, *k*_nr_ is the non-radiative rate. Both fluorescence quantum yield and lifetime will differ when considering the effect of metal. The total radiative deactivation rate will be written as Γ + Γ*_m_*, where Γ*_m_* corresponds to the radiative deactivation rate close to metal. So, the quantum yield and lifetime are modified as Equation (3) and (4):

(3) $$Q=\frac{\Gamma +\Gamma_{m}}{\Gamma +\Gamma_{m}+k_{nr}}$$

(4) $$\tau =\frac{1}{\Gamma +\Gamma_{m}+k_{nr}}$$

The fluorescence lifetime becomes shorter and the quantum yield is larger. Thus, fluorescence intensity is increased. The theoretical calculation matches the experimental results [32, 78]. Thirdly, the metallic nanostructures scattering affects the coupling efficiency of the fluorescence emission to the far field [79-80]. By regulating the plasmon resonance band to the fluorophore emission wavelength, a fluorescence enhancement can be obtained.

The metal nanostructure enhancement can only be observed at the suitable conditions. If the distance between the metal and fluorophore is less than 10 nm, the FRET will occur [81-82]. In this case, the incident light first excites the fluorophores, and then the excited fluorophores transfer the energy to the metal, resulting in nonradiative release [83]. Some experimental results also have shown that quenching exists in distances up to 100 nm [84]. This has been explained by the far field resonance interaction by the coupling of the excited fluorophores to the metal [79].

### 7.2 Metallic Nanostructures for NIRF Enhancement

A number of metallic nanostructures have been developed as the enhancement substrates for higher intensity of NIRF dyes, such as the silver island films [32, 85], silver colloid assembled films [86], silver nanorods [87], silver nanoplates [88], gold nanoshells [80], *etc.* A silver island of 100 ∼ 500 nm in diameter enhanced a modified NIR dye molecule (ICG-HSA) by 20-fold [32]. The enhancement mechanism is to increase ICG quantum yield and decrease its deactivation time when ICG is at an appropriate distance to silver island film. Halas *et al.* [80] used the gold nanoshells as the substrate to enhance ICG by adjusting the surface plasmon band close to ICG excitation and emission bands. A 50-fold fluorescence enhancement was achieved when the surface plasmon resonance band was tuned to the emission band of ICG. While an enhancement factor of only 15 was obtained when the surface plasmon resonance band overlapped with the ICG excitation band. This enhancement was attributed to the scattering efficiency of the metal nanoshell coupled with the fluorescence emission in the far field. The various metal enhancements have greatly improved the detection sensitivity of NIRF dye molecules in bioanalyses.

## Conclusions

Different types of NIRF materials have been developed. In general, NIRF dye molecules are traditional fluorescent labeling reagents in bioimaging and bioanalysis. The emerging nanomaterials provide NIRF a promising new direction. QDs and SWNTs have demonstrated great potential as photostable and highly intensive NIRF tags for the determination of biological targets. Meanwhile, doping a large number of NIRF molecules into a nanomatrix to produce an intense fluorescence nanoprobe is attractive since it can fully use the developed NIRF dye molecules to generate a wide variety of NIRF nanomaterials. To further improve the fluorescence intensity, metallic nanostructures are combined with NIRF materials for sensitive determinations of trace amounts of targets. Each type of NIR material brings its own strengths and weaknesses to the table. Pulling from the strengths of each, novel and highly sensitive NIRF probes can be developed. Two major advantages in NIR region, low background signal and deep penetration of radiation, providing NIRF probes broad applications in the biological and biomedical field.
